# Editorial: New frontiers in “bladder sparing” treatments for high risk NMIBC

**DOI:** 10.3389/fonc.2022.1118940

**Published:** 2023-01-11

**Authors:** Rodolfo Hurle, Massimo Lazzeri

**Affiliations:** Department of Urology, IRCCS Humanitas Clinical and Research Hospital, Rozzano, Italy

**Keywords:** bladder cancer, treatment, biomarker, imaging, MRI

Bladder cancer (BC) accounts for approximately 549000 new cases worldwide and 200000 deaths per year (1). The scenario of BC is changing rapidly and emerging questions on the pathology and molecular aspects, the role of imaging, and innovative bladder-sparing treatments are relevant to contemporary clinical practice.

High-risk non-muscle-invasive bladder cancer (HR-NMIBC) is notoriously difficult to treat and represents a challenge even for the most skilled urologists. Its recurrence rate remains high and the progression to muscle-invasive bladder cancer (MIBC) is not negligible (2). While radical cystectomy (RC) is the standard of care for MIBC, with or without neoadjuvant chemotherapy [www.uroweb.org; www.auanet.org], it is also proposed as “early cystectomy” in patients with BCG failure, very high-risk NMIBC, and after the failure of alternative conservative treatments (3). However, considering the morbidity of RC and its impact on patient QoL, most patients tend to reject RC and face the risk of disease progression, late salvage RC, and increased risk of death. There is a stringent “unmet clinical need” to preoperatively stratify the risk of progression and correctly stage the disease, primarily identifying lymph node metastasis, and offering personalized medicine. New insights raise more questions. Novel methods of treatment bring their own challenges, and several important problems remain to be addressed. In this Research Topic, the authors discuss clinical practice advances in patients’ stratification and management through a combination of clinical and pathological characteristics under radio-oncological, urological, and oncological frameworks.

The clinical staging and assessment of lymph node involvement in HR-NMIBC patients are mandatory in the decision-making process. The local extent of bladder cancer is important for prognosis and assessment of response to treatment. CT assessment of the detrusor muscle infiltration is poor, and a new VI-RADS score, which uses multiparametric MRI to assess the probability of the presence or absence of MIBC, has recently been suggested (4). However, it is not yet clear whether MRI should be reserved for the diagnostic work-up of HR-NMIBC to discriminate those that would benefit from surgery, neoadjuvant chemotherapy, or to be treated with new bladder-sparing therapies or trimodal therapy.

American and European guidelines recommend computed tomography (CT) or magnetic resonance imaging (MRI) to stage BC. They are anatomy-based imaging modalities that assess lymph node size and morphology. Positron emission tomography (PET), which uses a molecular tracer, has been considered an alternative as it also identifies metastases in normal-sized LN thanks to the specific tracer. Standard [18F]-FDG PET/CT has been reported to be more sensitive than CT and MRI, but its usefulness in BC loco-regional staging is constrained by inherent limitations. Recently, our group investigated the pre-operative diagnostic accuracy of modified [18F]-FDG PET/CT with diuretic administration (furosemide 20mg/2ml i.v.) and delayed acquisition, considering pathological data of HR or very HR (VHR) NMIBC patients who underwent radical cystectomy (RC) and concomitant pelvic lymph node dissection (LND). Preliminary unpublished data revealed a sensitivity of 62.5%, specificity of 57.1%, PPV of 62.5%, NPV of 57.1%, and an accuracy of 60.0% for the prediction of LN metastasis (**Figure 1**).

**Figure 1:**
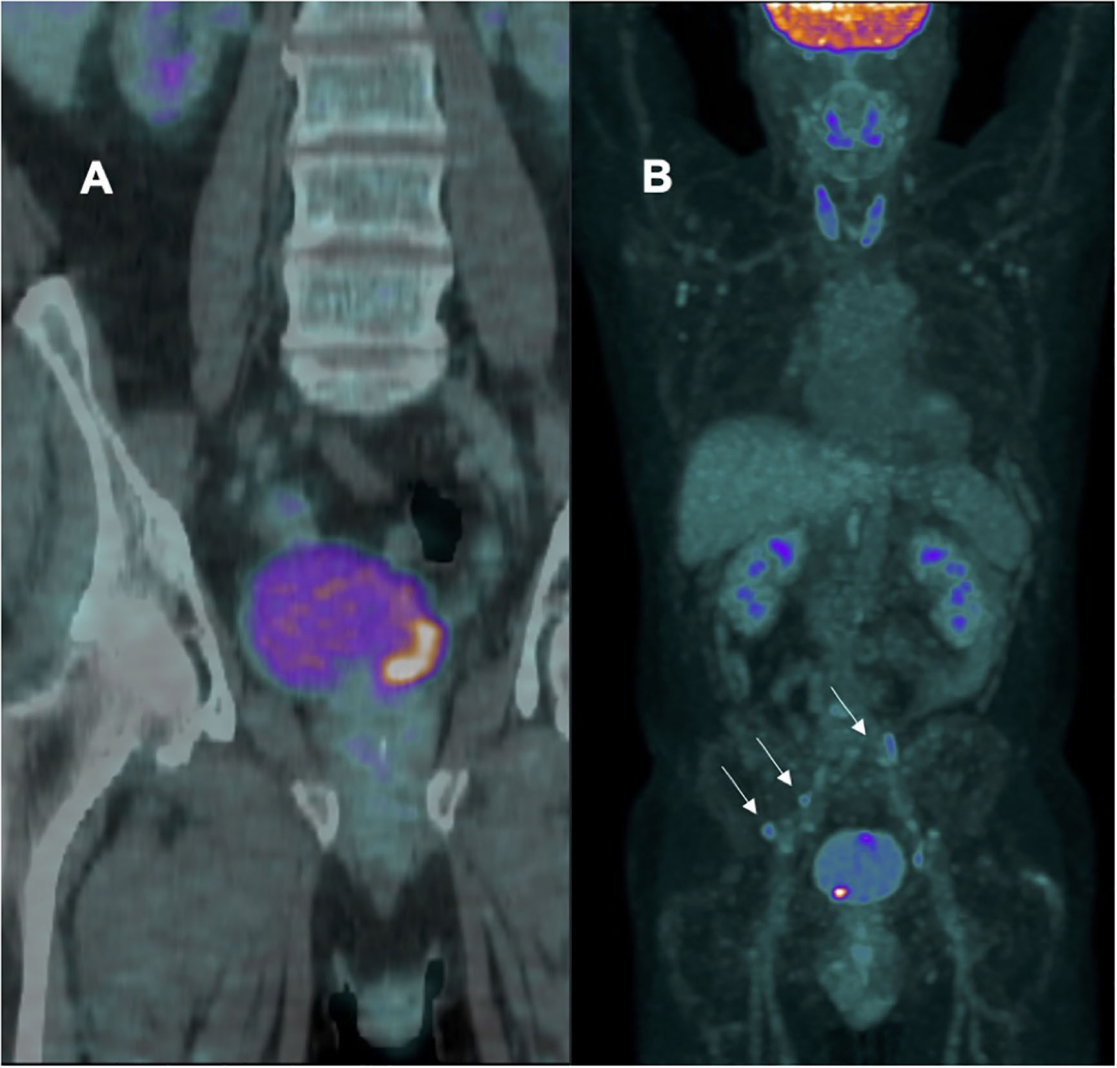
**(A)** patient with negative lymph node; **(B)** patient with multiple positive bilateral iliac lymph node (arrows).

In the last decade, the molecular spectrum of BC has been extensively studied. Several authors found that molecular subtypes correlate to cancer behavior and suggest specific sequences of treatment (5). Six consensus classes have been defined (luminal papillary, luminal non-specified, luminal unstable, stroma rich, basal squamous, and neuroendocrine-like) to gain advantages in prognostic and predictive counseling and to offer personalized medicine (6). Researchers are focusing on new methods of reporting pT1 subclassification (an important prognostic factor for progression to MIBC), and the recommendation for the routine use of molecular markers to assist in prognosis or to guide therapy will be soon considered in clinical practice (7).

Over the past 10 years, several important advances in the diagnosis and management of NMIBC have emerged and new achievements have resulted in better overall outcomes for patients. The current Research Topic contributes to better management of NMIBC. Patients’ selection, risk stratification, guiding treatments, and precision medicine will mean that patients with HR-VHR NMIBC will receive the best therapies, reaching the best outcomes with fewer toxicities and improved quality of life.

## Author contributions

All authors listed have made a substantial, direct, and intellectual contribution to the work and approved it for publication.
